# Association of Reduced Type IX Collagen Gene Expression in Human Osteoarthritic Chondrocytes With Epigenetic Silencing by DNA Hypermethylation

**DOI:** 10.1002/art.38774

**Published:** 2014-10-26

**Authors:** Kei Imagawa, María C de Andrés, Ko Hashimoto, Eiji Itoi, Miguel Otero, Helmtrud I Roach, Mary B Goldring, Richard O C Oreffo

**Affiliations:** 1 University of Southampton Medical School, Southampton, UK, and Tohoku University Graduate School of MedicineSendai, Japan; 2 University of Southampton Medical SchoolSouthampton, UK; 3 Tohoku University Graduate School of MedicineSendai, Japan; 6 Hospital for Special Surgery and Weill Cornell Medical CollegeNew York, New York; 4 Tohoku University Graduate School of MedicineSendai, Japan; 5 Hospital for Special Surgery, Weill Cornell Medical CollegeNew York, New York

## Abstract

**Objective:**

To investigate whether the changes in collagen gene expression in osteoarthritic (OA) human chondrocytes are associated with changes in the DNA methylation status in the *COL2A1* enhancer and *COL9A1* promoter.

**Methods:**

Expression levels were determined using quantitative reverse transcription–polymerase chain reaction, and the percentage of DNA methylation was quantified by pyrosequencing. The effect of CpG methylation on *COL9A1* promoter activity was determined using a CpG-free vector; cotransfections with expression vectors encoding SOX9, hypoxia-inducible factor 1α (HIF-1α), and HIF-2α were carried out to analyze *COL9A1* promoter activities in response to changes in the methylation status. Chromatin immunoprecipitation assays were carried out to validate SOX9 binding to the *COL9A1* promoter and the influence of DNA methylation.

**Results:**

Although *COL2A1* messenger RNA (mRNA) levels in OA chondrocytes were 19-fold higher than those in the controls, all of the CpG sites in the *COL2A1* enhancer were totally demethylated in both samples. The levels of *COL9A1* mRNA in OA chondrocytes were 6,000-fold lower than those in controls; 6 CpG sites of the *COL9A1* promoter were significantly hypermethylated in OA patients as compared with controls. Treatment with 5-azadeoxycitidine enhanced *COL9A1* gene expression and prevented culture-induced hypermethylation. In vitro methylation decreased *COL9A1* promoter activity. Mutations in the 5 CpG sites proximal to the transcription start site decreased *COL9A1* promoter activity. Cotransfection with SOX9 enhanced *COL9A1* promoter activity; CpG methylation attenuated SOX9 binding to the *COL9A1* promoter.

**Conclusion:**

This first demonstration that hypermethylation is associated with down-regulation of *COL9A1* expression in OA cartilage highlights the pivotal role of epigenetics in OA, involving not only hypomethylation, but also hypermethylation, with important therapeutic implications for OA treatment.

Collagen accounts for two-thirds of the dry weight of adult articular cartilage and confers upon the tissue the capacity to withstand tensile and shear forces. Within articular cartilage, type II collagen is the dominant collagen, accounting for more than 90% of the total collagen. The material strength of articular cartilage is dependent on the extensive cross-linking of the collagen and the zonal differences in fibrillar architecture, which depend on tissue depth (1). Collagen gene mutations account for a family of spondyloepiphyseal dysplasias, which are associated in most cases with early-onset osteoarthritis (OA) (2).

Type IX collagen is a fibril-associated collagen with interrupted triple helix (FACIT) that is proposed to stabilize the fibrillar and proteoglycan networks via lateral association with type II and type XI collagen (3), although only 1–5% of the total collagen of mature cartilage consist of this type of collagen. In contrast, mice that lack *Col9a1* develop normally but display OA-like cartilage degradation in the knee joints of older animals (4). Furthermore, the deficiency of *Col9a1* chains leads to a functional knockout of all polypeptides of type IX collagen, even though *Col9a2* and *Col9a3* are transcribed normally (5). In humans, *COL9A1* has been identified as a susceptibility locus for OA (6–8), where mutations predispose to OA but do not account for most cases. These studies indicate that type IX collagen is important for the formation of a stable collagen network and for the maintenance of cartilage organization and integrity. Reduced levels of type IX collagen in the matrix may render the cartilage more susceptible to damage by mechanical forces; thus, reduced *COL9A1* expression could ultimately contribute to the pathogenesis of human OA.

Gene expression is regulated by both epigenetic and nonepigenetic mechanisms, leading to up-regulation or down-regulation of genes that are part of the expression repertoire of a specific somatic cell type. Epigenetics refers to changes in gene expression without changes in the DNA sequence. The predominant epigenetic mechanisms are DNA methylation, histone modifications, and changes in higher-order chromatin structure. Nonepigenetic gene regulation is short-term, and expression reverts upon withdrawal of the relevant factors. In contrast, epigenetic regulation involves long-term silencing of all genes that are not normally expressed by a specific cell type. This silencing is important for genomic stability, and changes in epigenetic status may be associated with disease (9).

Methylation of genomic DNA represents a significant mechanism for regulating tissue-specific gene expression. We have previously shown that loss of DNA methylation underlies the aberrant expression of a number of catabolic genes in OA chondrocytes, including *MMP* genes, *ADAMTS4*, *IL1B*, and *NOS2* (10–14). Furthermore, the DNA methylation status of the *MMP13* promoter determines the levels of gene transcription and *MMP13* messenger RNA (mRNA) in chondrocytes (15,16). However, it remains unclear whether there is a role for either hypomethylation or hypermethylation in the silencing of anabolic genes associated with OA. Interestingly, a positive correlation between age and bone morphogenetic protein 7 (*BMP7*) methylation status in chondrocytes isolated from normal articular cartilage was reported (17), while hypermethylation of several CpG sites of superoxide dismutase 2 (SOD2) was found in OA cartilage, which correlated with decreased expression of the *SOD2* gene (18). Recent studies have shown the association of changes in DNA methylation status with the expression of the OA-associated gene *GDF5* (19), and increased DNA methylation and altered histone modification were observed in a study of the epigenetic status of the *SOX9* promoter (20).

Zimmermann and collaborators (21) have previously observed that the CpG island around the transcription start site (TSS) of the *COL2A1* promoter was completely demethylated in human articular chondrocytes, mesenchymal stem cells (MSCs), and MSC-derived chondrocytes independently of *COL2A1* gene expression. Levels of DNA methylation of the CpG sites in the 309-bp enhancer region, which is required for *COL2A1* transcription (21,22), had not been studied, however. We therefore examined the CpG methylation profile of the *COL2A1* enhancer region for a potential functional linkage to *COL2A1* gene expression.

It is known that the *COL9A1* promoter region from −846 bp to the TSS is important for its transcriptional regulation (23), and although there are 8 CpG sites in this promoter sequence, the effect of methylation on *COL9A1* transcription remains undetermined. In addition, the *COL9A1* promoter is known to have 5 SOX9-binding sites and 1 hypoxia inducible factor 1α (HIF-1α)/HIF-2α–binding site. SOX9, a transcription factor that is pivotal in chondrogenic differentiation, activates *COL9A1* gene expression (23). However, the role of CpG methylation in *COL9A1* transactivation by SOX9 remains unknown.

HIF-1α acts as a survival factor by enhancing extracellular matrix (ECM) synthesis (24) and inhibiting apoptosis (25). In contrast, HIF-2α is a catabolic regulator of OA cartilage destruction (26), and the binding and transactivation of *MMP13* by HIF-2α were shown to be inhibited by methylation of a specific CpG site in the proximal promoter (15).

The aim of the present study was to investigate whether the changes in collagen gene expression in human OA chondrocytes are associated with changes in the DNA methylation levels in the *COL2A1* enhancer and *COL9A1* promoter.

## Materials and Methods

### Chondrocyte isolation

Human chondrocytes were isolated from the articular cartilage of femoral heads obtained at the time of surgery for total hip replacement (12 OA patients [4 men and 8 women], with a mean ± SD age of 72.4 ± 7.9 years) or for femoral neck fracture (10 control subjects [2 men and 8 women], with a mean ± SD age of 79.2 ± 5.8 years), as previously described (27). Permission of the Local Ethics Committee and consent of the patients were obtained prior to this study.

### Chondrocyte culture

Only “non-OA chondrocytes” from the deep zone of tissues obtained from the patients with femoral neck fracture were used (27). Isolated chondrocytes were divided into 3 groups: noncultured (preculture), cultured without treatment (control culture), and cultured using 2 μ*M* 5-azadeoxycytidine (5-aza-dC). Prior to treatment, chondrocytes were cultured for 48 hours at 37°C at a density of 2–4 × 10^5^ cells in Dulbecco's modified Eagle's medium (DMEM)/F-12 medium supplemented with 5% fetal calf serum, 1% insulin–transferrin–selenium, 100 units/ml of penicillin, 100 μg/ml of streptomycin, and 100 μg/ml of ascorbic acid in an atmosphere of 5% CO_2_.

For samples cultured with 5-aza-dC, the histone deacetylase inhibitor trichostatin A (TSA; 300 n*M*) was added at the first treatment to facilitate access of 5-aza-dC, a cytidine analog that impedes the proper activity of DNA methyltransferase 1 (28). The media were changed twice weekly, and the primary cultures were maintained for 5 weeks until the samples reached confluence.

### DNA and RNA extraction

Total RNA and genomic DNA were extracted simultaneously from each sample with an AllPrep DNA/RNA Mini kit (Qiagen) according to the manufacturer's instructions. RNA was immediately reverse-transcribed with avian myeloblastosis virus reverse transcriptase and both oligo(dT)_15_ and random primers (29).

### Quantitative reverse transcription–polymerase chain reaction (qRT-PCR) analysis

Relative quantification of gene expression in total RNA extracts was performed with an ABI Prism 7500 detection system (Applied Biosystems). Reactions were performed in triplicate, with GAPDH as the internal control. Messenger RNA expression was quantified according to the 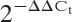 method, as previously described (9). Primer information is available upon request.

### DNA methylation analysis by bisulfite pyrosequencing

Bisulfite treatment of the genomic DNA of each sample was performed using an EZ DNA Methylation-Gold kit (Zymo Research) according to the manufacturer's instructions, as previously described (14).

**Figure 1:**
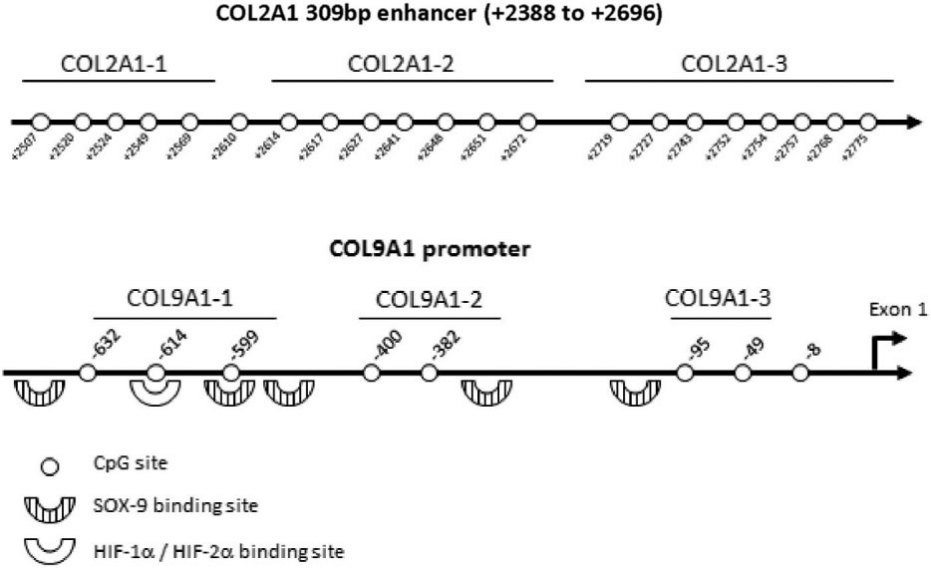
Features of the 309-bp *COL2A1* enhancer element (top) and the *COL9A1* promoter region (bottom). HIF-1α = hypoxia inducible factor 1α.

The percentage of DNA methylation in the *COL2A1* enhancer or the *COL9A1* promoter was quantified using a PyroMark MD system (Qiagen) according to the manufacturer's instructions. Primer information is available upon request from the corresponding author. Three primer sets were designed for each sequence of the *COL2A1* enhancer and *COL9A1* promoter to encompass the CpG sites of interest (Figure 1).

### COL9A1-Luciferase constructs

The human *COL9A1* proximal promoter region was PCR amplified from the genomic DNA isolated from human chondrocytes. The original sequences of the primers were derived from descriptions in the literature (23). The 5′ additional sequences of forward and reverse primers were modified to contain *Spe* I (construct 1 [C1]), *Bam* HI (C2), *Hind* III (C3), and *Nco* I sites to facilitate cloning. The PCR-generated promoter fragments (976 bp) contained the regions spanning −846 to +130 bp (C1), −565 to +130 bp (C2), and −169 to +130 bp (C3) relative to the TSS in the human *COL9A1* gene sequence. The promoter regions were cloned into the respective sites of the pCpG-free luciferase reporter vector constructed as described in the literature (15,30). After constructing the pCpG-free-Luc-COL9A1 vectors (C9–wild-type [C9-WT; C1], C2, and C3), pCpG-free-Luc-COL9A1-M1, M2, and M3 vectors were also produced with mutations in the CpG sites (see below). Primer information is available upon request from the corresponding author. In all cases, the correct insertion of constructs was determined by sequencing analysis.

### In vitro methylation of plasmid DNA

Methylated plasmids were generated by incubating 1 μg of plasmid DNA with 4 units/μl of the CpG methyltransferase, M.*Sss* I (New England BioLabs), according to the manufacturer's instructions. Complete methylation was verified by plasmid DNA bisulfite modification and pyrosequencing using specific primers.

### Transient transfection and luciferase assays

Cells of the immortalized human chondrocyte line C28/I2 (31) were seeded at a density of 30,000/well, cultured overnight in DMEM, and transfected with a mixture of 500 ng of luciferase reporter vector and 1 ng of pRL-SV40 vector, using TurboFect in vitro transfection reagent (Fermentas) and FuGene HD (Promega) according to manufacturers' recommendations. Transfected C28/I2 cells were cultured for 24–48 hours prior to harvest. Cell lysates were assayed for firefly and *Renilla* luciferase activity using a Dual-Luciferase Reporter Assay system (Promega) on a Varioskan Flash reader (Thermo). Firefly luciferase activity for each transfection was normalized against *Renilla* luciferase activity. For the cotransfection assays, 500 ng of SOX9-pcDNA vector (32) and 500 ng of the HA-HIF-1α-pcDNA3 vector (Addgene plasmid 18949) or the HA-HIF-2α-pcDNA3 vector (Addgene plasmid 18950) (33) were used. The pcDNA3 vector (Invitrogen) served as a negative control.

### Chromatin immunoprecipitation (ChIP) assay

A ChIP-IT Express Enzymatic kit (Active-Motif) was used to perform ChIP assays according to the manufacturer's instructions, as described elsewhere (15). Briefly, C28/I2 cells were cotransfected with unmethylated or methylated pCpG-free-Luc-COL9A1 vector (−846 to +130 bp) and the expression vector encoding SOX9 using FuGene HD (Promega). At 48 hours following transfection, precleared chromatin was stored as assay input or was incubated overnight at 4°C with 5 μg of rabbit anti-SOX9 antibody (Abcam) or normal rabbit IgG (Cell Signaling Technologies). After reverse cross-linking and purification, the final DNA preparations were subjected to qPCR analysis using 5 μl of the eluted DNA. For analysis, the C_t_ of each sample was normalized to the C_t_ of the input sample. Specific primers for each binding site were designed (primer information is available upon request from the corresponding author).

### Statistical analysis

Expression and percentage methylation data were analyzed using Wilcoxon's signed rank test. The data for COL9A1 luciferase reporter assay were analyzed using analysis of variance with a post hoc *t*-test to check the differences between two groups. *P* values less than 0.05 were considered significant.

## Results

### Features of the *COL2A1* enhancer and the *COL9A1* promoter regions

The structures of the 309-bp enhancer region of *COL2A1* and the promoter region of *COL9A1* are shown in Figure 1. The 309-bp enhancer region of *COL2A1* has 21 CpG sites, and the *COL9A1* promoter has 8 CpG sites. The +2610-bp CpG site in the *COL2A1* enhancer and −8-bp CpG site in the *COL9A1* promoter were not analyzed. The *COL9A1* promoter construct contains 1 HIF-1α/HIF-2α binding site and 5 SOX9 binding sites.

### No relationship between increased *COL2A1* mRNA levels in OA chondrocytes and DNA methylation changes in the 309-bp *COL2A1* enhancer

**Figure 2:**
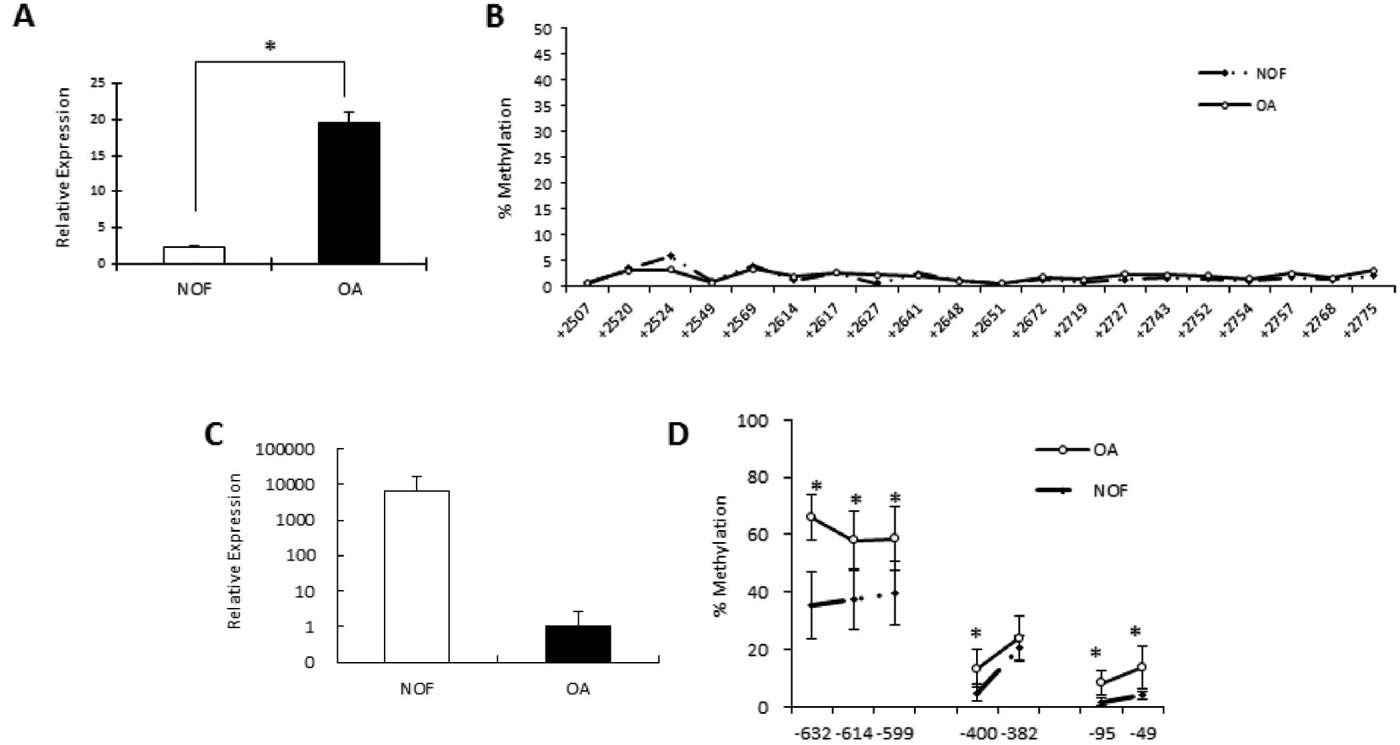
Correlation between hypermethylation at specific *COL9A1* proximal promoter CpG sites in chondrocytes from patients with osteoarthritis (OA) and reduced levels of gene expression in OA disease. A and C, Relative expression of mRNA for *COL2A1* (A) or *COL9A1* (C) in noncultured primary chondrocytes from 10 patients with femoral neck fracture (NOF; controls) and 12 patients with OA, as determined by quantitative reverse transcription–polymerase chain reaction analysis and normalized against GAPDH. B and D, Percentage DNA methylation of the indicated CpG sites on the *COL2A1* (B) and *COL9A1* (D) proximal promoters in chondrocytes from the 10 femoral neck fracture patients and 12 OA patients, as determined by bisulfite pyrosequencing analysis of the same samples. Values are the mean ± SD of triplicate (A and C) or duplicate (B and D) determinations for each sample. Samples tested in B and D are the same as those in A and C, respectively. ∗ = *P* < 0.05 for OA versus controls.

Chondrocytes were isolated from cartilage, and extracts of total RNA were immediately prepared for qRT-PCR analysis. *COL2A1* mRNA levels in chondrocytes derived from OA patients were 9-fold higher than those in control chondrocytes (Figure 2A).

The percentage of DNA methylated CpG sites in the 309-bp enhancer of *COL2A1* was quantified using pyrosequencing to determine whether the increase in *COL2A*1 expression in OA chondrocytes was associated with epigenetic unsilencing by DNA demethylation (Figure 2B). The 309-bp sequence contains 21 CpG sites; 3 primer pairs were designed to cover the 21 CpG sites in the *COL2A1* enhancer (Figure 1). All CpG sites analyzed in the 309-bp *COL2A1* enhancer were almost completely demethylated in both femoral neck fracture and OA samples.

### Association between the loss of *COL9A1* gene expression in OA chondrocytes and hypermethylation of CpG sites in the *COL9A1* promoter

High levels of *COL9A1* expression were observed in control samples, although considerable patient-to-patient variation was noted. Interestingly, *COL9A1* mRNA levels in OA chondrocytes were significantly different and were determined to be 6,200-fold lower than those in chondrocytes from patients with femoral neck fracture (Figure 2C). Potential differences between expression profiles may exist when comparing results from isolated cells with those obtained from RNA isolated directly from articular cartilage.

To determine whether the loss of *COL9A1* expression in OA cartilage was associated with DNA hypermethylation, DNA methylation levels were quantified by bisulfite pyrosequencing. The *COL9A1* promoter contains 8 CpG sites in the proximal promoter, and 3 primer pairs were designed to cover the CpG sites located at −632, −614, and −599 bp, −400 and −382 bp, and −95 and −49 bp. At the CpG sites closest to the TSS, negligible DNA methylation was observed (<4%) in femoral neck fracture samples, but it was 8% to 14% higher in OA samples. At the −400-bp and −382-bp sites, the overall percentage methylation was higher than at the CpG closest to the TSS, at 5% and 20%, respectively, in femoral neck fracture samples and 13% and 24%, respectively, in OA samples. Significant differences in the percentage methylation were observed between control and OA patient samples at −400 bp, although not at −382 bp (Figure 2D). In contrast, enhanced methylation at CpG sites −632, −614, and −599 bp were observed with, on average, ∼40% methylated sites in control samples and ∼60% in OA chondrocytes. A significant reciprocal trend between the percentage of DNA methylation and mRNA expression was observed, and the magnitude of hypermethylation in OA samples was found to be greatest between CpG sites −632 bp and −599 bp (Figure 2D).

### Association between loss of *COL9A1* expression in culture and hypermethylation

**Figure 3:**
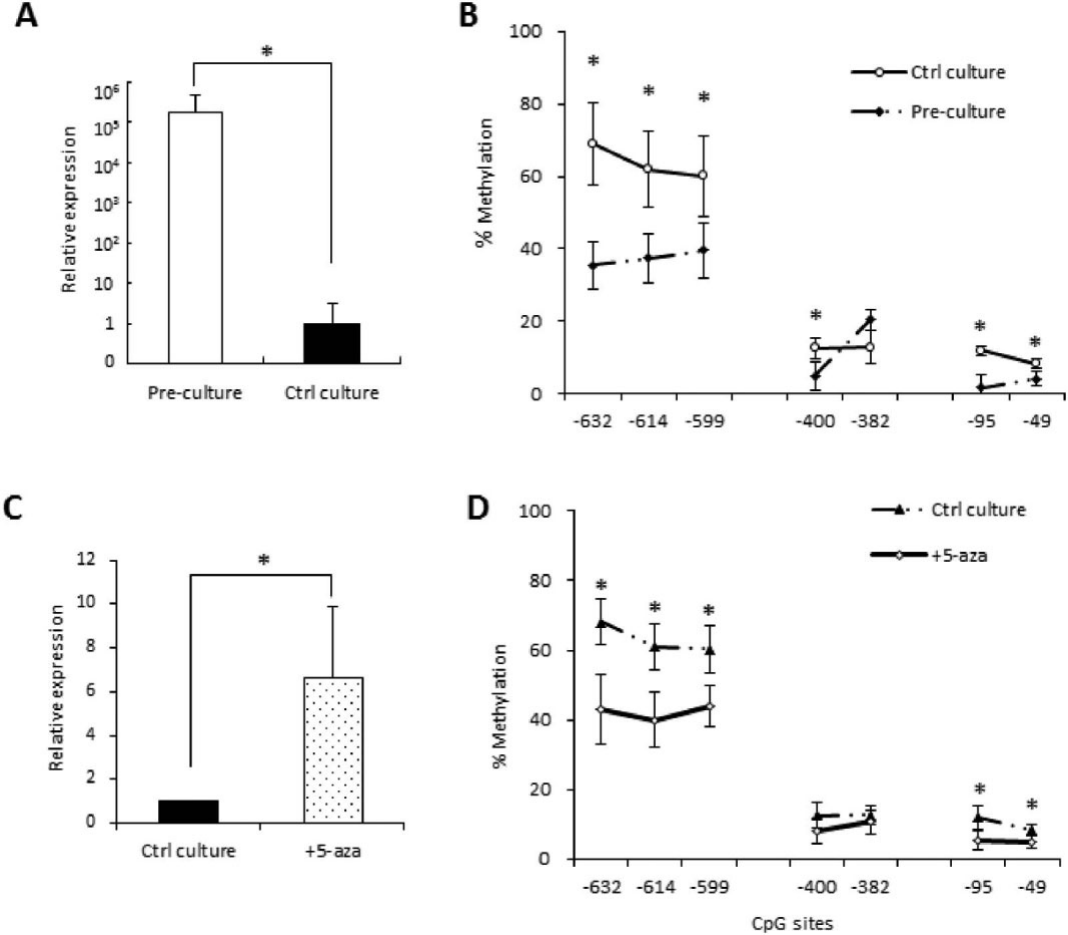
Correlation of culture conditions and CpG demethylation in vitro with decreased and enhanced levels of *COL9A1* gene expression in chondrocytes obtained from 10 patients with femoral neck fracture. A and C, Relative mRNA expression of *COL9A1* in preculture chondrocytes compared with control culture chondrocytes (A) as well as in control culture chondrocytes compared with chondrocytes cultured for 5 weeks in 5-azadeoxycytidine (5-aza-dC [+5-aza]; with trichostatin A added once to facilitate access of 5-aza-dC) (C), as determined by quantitative reverse transcription–polymerase chain reaction analysis. B and D, Percentage DNA methylation of the indicated CpG sites in the *COL9A1* promoter in preculture chondrocytes compared with control culture chondrocytes (B) as well as in control culture chondrocytes compared with chondrocytes cultured for 5 weeks in 5-aza-dC (D), as determined by bisulfite pyrosequencing analysis of the same samples. Values are the mean ± SD of triplicate (A and C) or duplicate (B and D) determinations for each sample (n = 10 preculture samples, n = 7 control culture samples, and n = 6 5-aza-dC–treated samples). ∗ = *P* < 0.05 for preculture or 5-aza-dC–treated samples versus control culture samples.

Since monolayer culture is known to affect the gene expression profile of chondrocytes (34), *COL9A1* mRNA levels were analyzed in preculture chondrocytes and compared with chondrocytes that had been cultured for 5 weeks (Figure 3A). Chondrocytes were obtained from patients with femoral neck fracture. The culture of chondrocytes proved detrimental to *COL9A1* expression, with significant loss of expression. Analysis of the percentage of DNA methylation of the *COL9A1* promoter demonstrated that the significant loss of *COL9A1* expression as a consequence of culture was associated with hypermethylation (Figure 3B).

### Enhanced *COL9A1* expression and prevention of culture-induced hypermethylation following 5-aza-dC treatment

We examined whether culture-induced hypermethylation of CpG sites in the *COL9A1* promoter could be ameliorated by 5 weeks of treatment with 5-aza-dC (trichostatin A was added once to facilitate access of 5-aza-dC). *COL9A1* mRNA levels in chondrocytes cultured with 5-aza-dC were ∼7-fold higher than those in control culture chondrocytes (Figure 3C). Significantly, the percentage methylation of the *COL9A1* promoter in these chondrocyte cultures was maintained at levels comparable to those in preculture chondrocytes (Figure 3D).

### Methylation-induced decrease in *COL9A1* promoter activity in vitro

**Figure 4:**
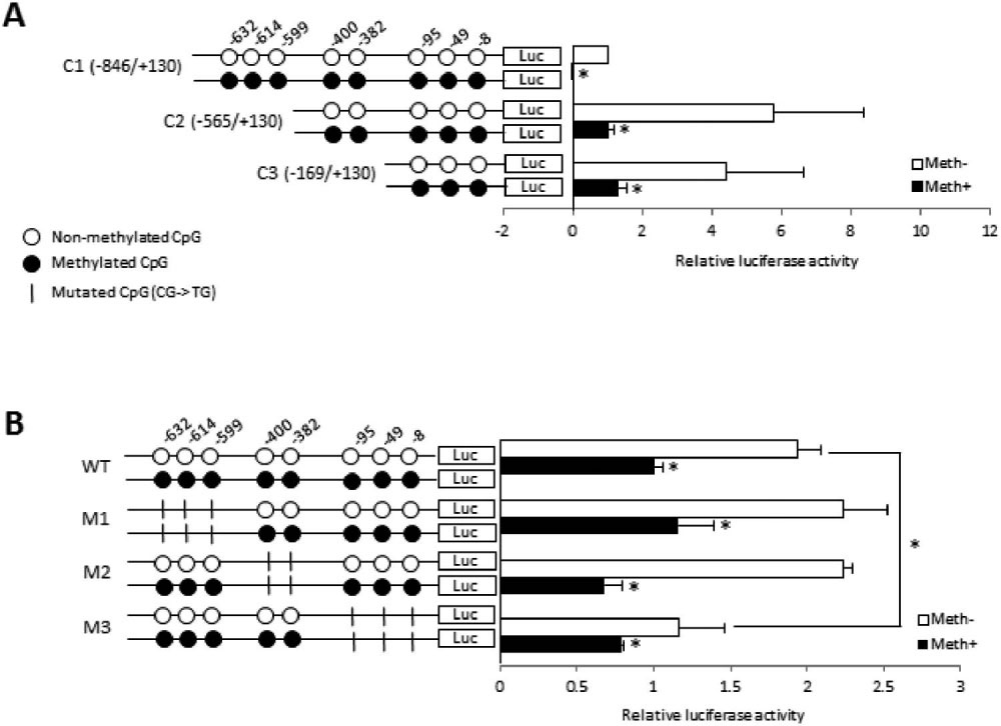
Effect of methylation of CpG sites in the proximal human *COL9A1* gene promoter on transcriptional activity. *COL9A1* promoter activity was analyzed using the luciferase (Luc) assay in C28/I2 chondrocytes transfected with either C1 (wild-type [WT]) or deletions from the 5′ ends of constructs C2 and C3 (A) or transfected with specific CpG mutations (M1, M2, and M3) (B), without (Meth−) or with (Meth+) CpG methylation treatment. Values are the mean ± SD of 3–5 independent experiments, each performed in triplicate. ∗ = *P* < 0.05 versus no methylation treatment or for the indicated comparison, by analysis of variance with post hoc *t*-test.

To determine the effect of methylation on *COL9A1* promoter activity, we used a luciferase reporter assay. C28/I2 chondrocytes were transfected with the wild-type *COL9A1* construct in the CpG-free vector (C9-WT) or with deletions from the 5′ end of constructs C2 (−565 to +130 bp) and C3 (−169 to +130 bp) and pRL-SV40 vector as an internal control and were then harvested for the luciferase assay. Methylation treatment significantly decreased the activities of the 3 constructs (Figure 4A). Interestingly, shorter constructs were significantly more active than WT, indicating that CpG sites closer to the TSS are crucial to *COL9A1* promoter activity.

### Association between mutations in the 3 CpG sites proximal to the TSS and decreased *COL9A1* promoter activity

To determine the critical CpG sites for *COL9A1* promoter activity, we compared the C9-WT and 3 vectors with mutations at different CpG sites (Figure 4B). Methylation decreased the *COL9A1* promoter activity in cells transfected with C9-WT or with any of the 3 vectors containing CpG mutations. Significantly lower activity was observed in cells transfected with C9-M3 as compared those transfected with C9-WT in their unmethylated forms, indicating that the 3 CpG sites proximal to the TSS are key for *COL9A1* promoter activity. The ratios of luciferase activities in unmethylated-to-methylated vectors were 1.9, 1.9, 3.3, and 1.5 in C9-WT, M1, M2, and M3, respectively, with a smaller ratio indicating a reduced effect of methylation on promoter activity.

### SOX9-induced enhancement of *COL9A1* promoter activity

**Figure 5:**
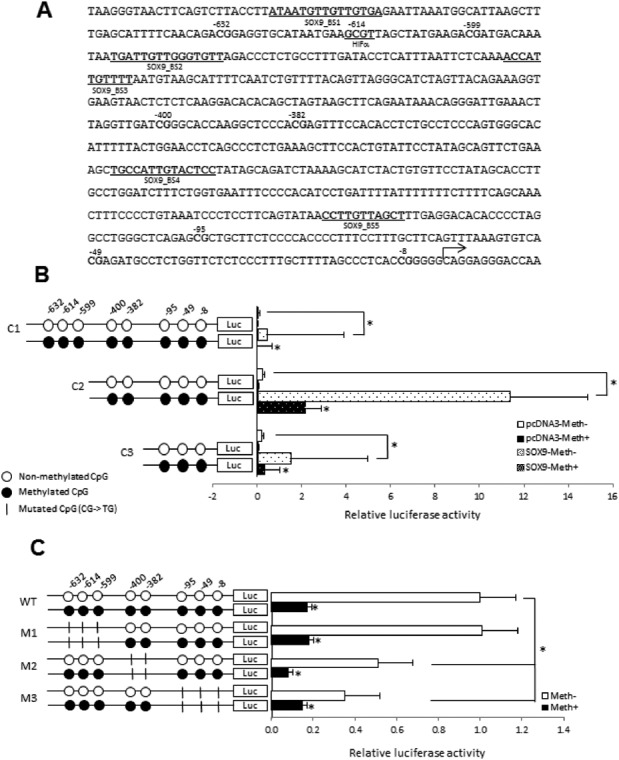
Impaired SOX9-driven *COL9A1* promoter transactivation in the presence of CpG methylation. A, Sequence of the proximal human *COL9A1* promoter. SOX9 putative binding sites (BS1–BS5), as well as the hypoxia inducible factor 1α (HIF-1α) binding site, are underlined. Positions of CpG sites are indicated (−652 bp to −8 bp). B, Different unmethylated (Meth−) and methylated (Meth+) *COL9A1* constructs were cotransfected with the empty control vector (pcDNA3) or with the SOX9 expression vector. C, Effect of CpG mutation on SOX9 cotransfection. Constructs and CpG mutations used for transfections are the same as those in Figure 4. Values in B and C are the mean ± SD of 3 independent experiments, each performed in duplicate. ∗ = *P* < 0.05 versus SOX9 no methylation treatment in B and versus no methylation treatment in C and for the indicated comparisons.

The sequence of the 5′-flanking region of the human *COL9A1* gene from −711 to +13 bp is shown in Figure 5A. The CpG positions and the 5 putative SOX9-binding sites (BS1–BS5) in the construct are highlighted.

To determine the effects of the transcription factors HIF-1α, HIF-2α (data not shown), and SOX9 on *COL9A1* promoter activity, the expression vector encoding each transcription factor or the pcDNA3 empty vector was cotransfected with C9-WT (C1), C9-C2, or C9-C3. Overexpression of either HIF-1α or HIF-2α had a negligible effect on the activities of all the constructs (data not shown). Consistent with previous work (23), SOX9 overexpression increased the activity of C9-WT and, especially C9-C2 and C9-C3, constructs that have 5 and 3 CpG sites, respectively, showing an increase in luciferase activity of ∼56-fold and ∼8.75-fold, respectively (Figure 5B). Interestingly, CpG methylation significantly attenuated this SOX9-mediated promoter activation in all constructs (Figure 5B).

### Requirement of 5 CpG sites proximal to the TSS for transactivation of *COL9A1* by SOX9

To determine which CpG sites within the *COL9A1* promoter were critical for SOX9 transactivation, each of the 4 COL9A1 vectors (C9-WT, M1, M2, and M3) was cotransfected with the pcDNA-SOX9 vector (Figure 5C). Mutation of the 3 CpG sites in the upstream region (C9-M1) had a negligible effect on transactivation of the *COL9A1* promoter by SOX9. In contrast, the mutations in the 5 CpG sites close to the TSS resulted in significant loss of *COL9A1* promoter activity. The ratios of SOX9-driven luciferase reporter activity in unmethylated-to-methylated vectors were 5.8, 5.6, 6.3, and 2.3 in C9-WT, M1, M2, and M3, respectively.

### CpG methylation–induced attenuation of SOX9 binding to the *COL9A1* promoter

**Figure 6:**
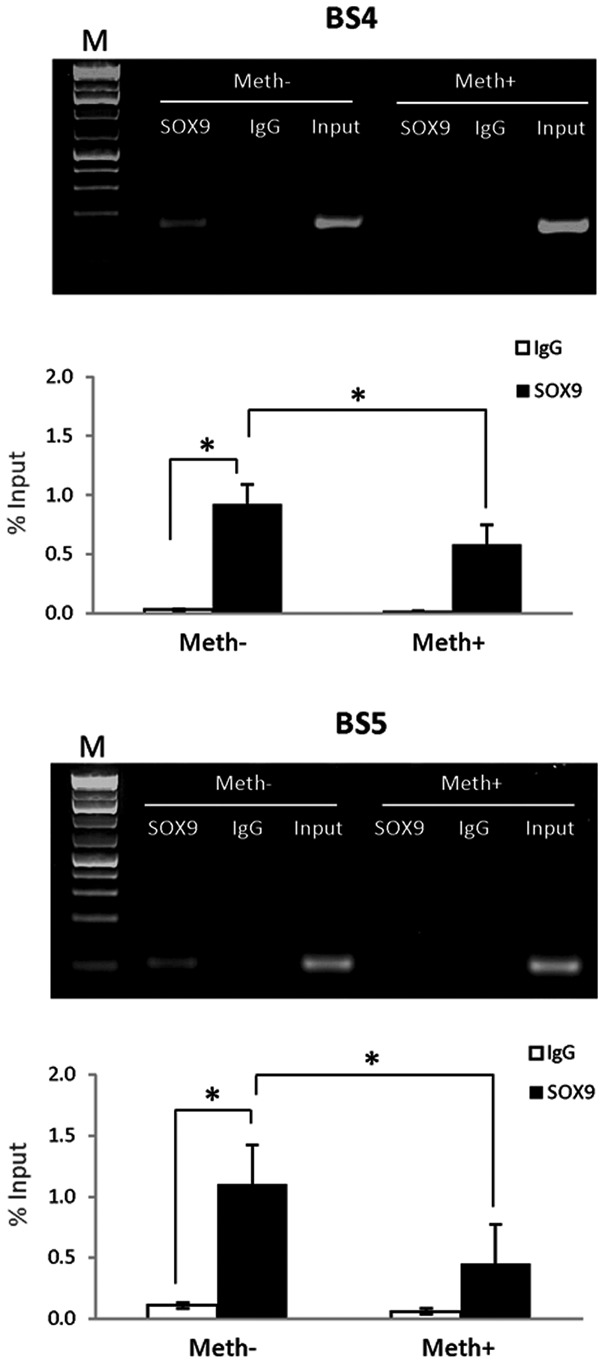
Attenuated SOX9 binding to the *COL9A1* promoter in the presence of CpG methylation. Chromatin immunoprecipitation assays were performed using cell lysates from C28/I2 cells that had been transfected with an unmethylated (Meth−) or a methylated (Meth+) wild-type (−846 to +130-bp) *COL9A1* promoter construct (Input) and the expression vector encoding SOX9. Binding of transcription factor to the human *COL9A1* promoter was analyzed by quantitative polymerase chain reaction using primers specifically bracketing SOX9-binding sites BS4 and BS5. The results were quantified and are shown as the percentage input. Values are the mean ± SD of 4 experiments and represent the fold-change versus IgG. ∗ = *P* < 0.05.

In order to analyze our results in more detail, we investigated whether the CpG methylation status directly affected SOX9 binding to the proximal *COL9A1* promoter in ChIP assays performed using C28/I2 chondrocytes cotransfected with unmethylated or methylated WT −846 to +130-bp *COL9A1* promoter constructs and expression vectors encoding SOX9. *COL9A1* promoter binding was analyzed with specific PCR primers that recognized only the transiently transfected promoter construct. In addition, primers were specifically designed to recognize each putative SOX9-binding site. ChIP assays revealed that methylation treatment significantly reduced SOX9 binding to the *COL9A1* proximal promoter, specifically to the putative binding sites BS4 and BS5 (Figure 6). These observations are consistent with our results showing decreased SOX9-driven COL9A1 transactivation after methylation treatment of the mutant *COL9A1* reporter constructs (Figure 5B).

## Discussion

Our current findings indicate that the increased *COL2A1* mRNA levels that we observed in OA chondrocytes do not correlate with changes in the methylation status, either at the promoter or the enhancer regions. This is contrary to our recent findings on the *NOS2* enhancer (14) and *MMP13* promoter (15). It is possible that in the absence of differential methylation, the increased expression of *COL2A1* observed in OA chondrocytes could be explained by the regenerative efforts of the cells to restore the ECM as an anabolic response within a degradative environment. Furthermore, the widespread expression of *COL2A1* in OA chondrocytes suggests a compensatory response to the absence of *COL9A1*. Our findings of increased *COL2A1* expression in OA are consistent with those observed by Ijiri and coworkers (35) and by Aigner and collaborators (36) in microarray analyses.

Interestingly, Sesselmann and colleagues (37) reported that DNA methylation of the promoter of *p21WAF1/CIP1* is not responsible for down-regulation of *p21WAF1/CIP1* mRNA in OA chondrocytes. Since *p21WAF1/CIP1* is an inhibitor of proliferation that is expressed in normal chondrocytes, it was postulated that down-regulation in OA, which is associated with increased cell division, may have an epigenetic component. Similarly, Poschl and colleagues (38) reported that DNA methylation is not a key component of the down-regulated expression of aggrecan, another major chondrocyte gene. None of these studies could demonstrate changes in the methylation status of genes related to OA phenotype.

It is likely that the crucial difference between our results lies in the promoter structure of the gene. Both aggrecan and the *p21* promoters did not show any difference in the DNA methylation status in their noncoding regions, which contain CpG islands that are absent from the *COL9A1* promoter. As shown by the Human Epigenome Project (39), genes with large numbers of CpG islands are, in general, not methylated in normal cells, irrespective of expression. Treatment with 5-aza-dC provided a useful tool with which to determine whether decreased methylation caused the activation of transcription in a specific gene (20). The current observations demonstrate that the low level of *COL9A1* mRNA in OA chondrocytes could be reversed through inhibition of DNA methylation. Our findings of decreased *COL9A1* expression in OA are consistent with recent proteomic (40) and microarray studies (41); however, other groups failed to observe this significant decrease in *COL9A1* expression on gene profiling of OA (36,42,43).

To determine the role of DNA methylation on the *COL9A1* promoter, we used a CpG-free vector (30) within a transfection assay in vitro. Site-directed mutations of the 8 CpG sites within the 976-bp *COL9A1* promoter in the construct revealed, for the first time, that *COL9A1* promoter activity is significantly decreased by CpG methylation in articular chondrocytes. We found significantly lower promoter activity when we transfected C9-M3, which lacks 3 CpG sites proximal to the TSS, as compared with C9-WT. Similarly, other investigators (44) have reported that effective gene suppression is observed only when promoters are methylated in the preinitiation domain.

Zhang and coworkers (23) reported that the −560 to −357-bp region of the *COL9A1* promoter is important for full *COL9A1* promoter activity in the rat chondrosarcoma cell line. In this current study, mutation of the −400 and −382-bp CpG sites in C9-M2 did not alter promoter activity. Thus, it can be assumed that the promoter activity of the −560 to −357-bp sequence is regulated by a mechanism other than CpG methylation within that region. In contrast, our study indicates that 3 CpG sites (−95, −49, and −8 bp) in the proximal promoter are also important for full *COL9A1* promoter activity.

SOX9 is a transcription factor that is essential for chondrogenesis (45) and indispensable for skeletogenesis (46,47). While some reports indicate no changes or increased expression of *SOX9* in early OA disease (48), OA is generally associated with down-regulation of *SOX9* expression (49). However, the lack of positive correlation between *SOX9* and *COL2A1* expression in adult articular chondrocytes suggests that while *SOX9* is essential for chondrogenesis (45) and normal and OA cartilage homeostasis, it is not the key regulator of the *COL2A1* promoter activity in human adult articular chondrocytes (50). Furthermore, Kim et al (20) recently reported that hip OA is associated with a change in the epigenetic status of the *SOX9* promoter, including increased DNA methylation and altered histone modifications. The cause–effect relationship between the epigenetic change in *SOX9* promoter and hip OA was not elucidated, however.

Taking into account the down-regulation of *SOX9* expression during chondrocyte dedifferentiation, its hypermethylated promoter in OA disease, and its methylation-dependent control of *COL9A1* transcription, it is conceivable that the OA environment associated with decreased *SOX9* expression and abnormal methylation patterns enhance the susceptibility of the *COL9A1* promoter to DNA methylation.

It has been shown that SOX9 enhances *COL9A1* promoter activity (23); however, the CpG sites in the *COL9A1* promoter required for transactivation by SOX9 remain unknown. Consistent with Zhang et al (23), our results indicate that methylation of the CpG sites in the *COL9A1* promoter attenuated the SOX9-mediated enhancement. Mutation analysis confirmed that the 5 CpG sites proximal to the TSS were responsible for the enhancement of *COL9A1* promoter activity by SOX9.

Using promoter mutation constructs it appears that the regions that are susceptible to methylation may be the same as those that are responsible for SOX9 binding and transcriptional activation. Interestingly, mutation does not completely abolish the effect of methylation, which suggests that methylation at multiple sites throughout the *COL9A1* promoter determines transcription. Consistent with Zhang and collaborators, enhanced promoter activity was observed after cotransfection of the C2 construct with SOX9; indeed, direct mutagenesis of −400 and −382 bp (M2) and/or −95, −49, and −8 bp (M3) showed the most significant decrease in the promoter activity.

This study is the first to show that in chondrocytes, CpG methylation of the *COL9A1* proximal promoter specifically impairs SOX9-driven promoter activation by altering SOX9 binding to DNA and that transactivation depends mainly on the DNA methylation status of 2 SOX/sex-determining region Y (SRY) binding sites (BS4 and BS5).

In contrast, cotransfection experiments carried out with HIF-1α and HIF-2α showed no significant changes, indicating that they are not the key transcription factors that modulate *COL9A1* promoter activity (data not shown). It is thus likely that other transcription factors intercalating between HIFs and *COL9A1* modulate function, and this will require further investigation.

In conclusion, epigenetic changes in OA involve hypomethylation and the consequent activation of aberrant, catabolic genes, as well as hypermethylation leading to silencing of at least 1 chondrocytic gene that contains sparse CpG sites at important regulatory domains. This is the first demonstration that hypermethylation is associated with down-regulation of *COL9A1* expression in OA, indicating the pivotal role of epigenetics in decreased anabolism in OA. Undoubtedly, other genes subject to epigenetic regulation remain to be identified in OA, and while changes in DNA methylation will not always explain the permanent alteration of gene expression in OA chondrocytes, our studies suggest that approaches that incorporate prevention or reversal of epigenetic changes offer significant therapeutic potential for OA in an increasing patient demographic.

## Author Contributions

All authors were involved in drafting the article or revising it critically for important intellectual content, and all authors approved the final version to be published. Dr. Oreffo had full access to all of the data in the study and takes responsibility for the integrity of the data and the accuracy of the data analysis.

**Study conception and design.** Imagawa, de Andrés, Hashimoto, Roach, Goldring, Oreffo.

**Acquisition of data.** Imagawa, de Andrés, Hashimoto.

**Analysis and interpretation of data.** Imagawa, de Andrés, Hashimoto, Itoi, Otero, Roach, Goldring, Oreffo.
